# Comparison of diagnostic criteria for acute kidney injury in critically ill children: a multicenter cohort study

**DOI:** 10.1186/s13054-022-04083-0

**Published:** 2022-07-07

**Authors:** Yuxian Kuai, Min Li, Jiao Chen, Zhen Jiang, Zhenjiang Bai, Hui Huang, Lin Wei, Ning Liu, Xiaozhong Li, Guoping Lu, Yanhong Li

**Affiliations:** 1 Department of Nephrology and Immunology, Children’s Hospital of Soochow University, Suzhou, Jiangsu Province China; 2 Pediatric Intensive Care Unit, Anhui Provincial Children’s Hospital, Hefei, Anhui Province China; 3 Pediatric Intensive Care Unit, Children’s Hospital of Soochow University, Suzhou, Jiangsu Province China; 4 Pediatric Intensive Care Unit, Xuzhou Children’s Hospital, Xuzhou, Jiangsu province China; 5 Pediatric Intensive Care Unit, Children’s Hospital of Fudan University, Shanghai, China; 6 Institute of Pediatric Research, Children’s Hospital of Soochow University, Suzhou, Jiangsu Province China

**Keywords:** Acute kidney injury, Consensus definition, Critically ill children, Serum creatinine

## Abstract

**Background:**

Substantial interstudy heterogeneity exists in defining acute kidney injury (AKI) and baseline serum creatinine (SCr). This study assessed AKI incidence and its association with pediatric intensive care unit (PICU) mortality under different AKI and baseline SCr definitions to determine the preferable approach for diagnosing pediatric AKI.

**Methods:**

In this multicenter prospective observational cohort study, AKI was defined and staged according to the Kidney Disease: Improving Global Outcome (KDIGO), modified KDIGO, and pediatric reference change value optimized for AKI (pROCK) definitions. The baseline SCr was calculated based on the Schwartz formula or estimated as the upper normative value (NormsMax), admission SCr (AdmSCr) and modified AdmSCr. The impacts of different AKI definitions and baseline SCr estimation methods on AKI incidence, severity distribution and AKI outcome were evaluated.

**Results:**

Different AKI definitions and baseline SCr estimates led to differences in AKI incidence, from 6.8 to 25.7%; patients with AKI across all definitions had higher PICU mortality ranged from 19.0 to 35.4%. A higher AKI incidence (25.7%) but lower mortality (19.0%) was observed based on the Schwartz according to the KDIGO definition, which however was overcome by modified KDIGO (AKI incidence: 16.3%, PICU mortality: 26.1%). Furthermore, for the modified KDIGO, the consistencies of AKI stages between different baseline SCr estimation methods were all strong with the concordance rates > 90.0% and weighted kappa values > 0.8, and PICU mortality increased pursuant to staging based on the Schwartz. When the NormsMax was used, the KDIGO and modified KDIGO led to an identical AKI incidence (13.6%), but PICU mortality did not differ among AKI stages. For the pROCK, PICU mortality did not increase pursuant to staging and AKI stage 3 was not associated with mortality after adjustment for confounders.

**Conclusions:**

The AKI incidence and staging vary depending on the definition and baseline SCr estimation method used. The modified KDIGO definition based on the Schwartz method leads AKI to be highly relevant to PICU mortality, suggesting that it may be the preferable approach for diagnosing AKI in critically ill children and provides promise for improving clinicians’ ability to diagnose pediatric AKI.

**Supplementary Information:**

The online version contains supplementary material available at 10.1186/s13054-022-04083-0.

## Background

Acute kidney injury (AKI) is an abrupt decline in renal function and is associated with increased adverse outcomes [1, 2]. The Kidney Disease: Improving Global Outcome (KDIGO) criterion originally used for the diagnosis of AKI in adults is commonly accepted [3, 4]. However, the high variability of serum creatinine (SCr) in children was not taken into account in the criterion designed for adults. To date, no definition has been shown to be superior, and no universal consensus exists regarding which definition to use in pediatrics [5–7].

Some studies have modified the KDIGO criterion by stipulating that the SCr increase should reach a concentration of at least 0.5 mg/dL for patients to be diagnosed with pediatric AKI [8, 9]. A multicenter study of hospitalized children in China proposed a new definition for pediatric AKI (pediatric reference change value optimized for acute kidney injury, pROCK), which was based on the concept that only an acute increase in SCr above the upper limit of normal variability represents a true decline in renal function [10]. The above two definitions combine the physiological characteristics of children and are stricter than the KDIGO criterion. However, they are less sensitive than KDIGO, and whether AKI diagnosed by the two definitions would be highly relevant to outcome remains elusive. Therefore, it is necessary to compare the KDIGO criterion with the later proposed definitions.

Another challenge in reporting pediatric AKI relates to the ascertainment of baseline SCr, which is often unavailable in hospitalized patients. Some studies conducted in pediatric populations calculated baseline SCr using the Schwartz equation assuming a glomerular filtration rate (GFR) of 120 mL/min/1.73 m^2^ [11], but some researchers suggested that using reference values (based on sex and age) for SCr as the baseline is a rational choice [12], and some studies accepted the first available value following admission as the baseline SCr (AdmSCr) [13]. In addition to the above methods, our research team has always adopted a measurement method (modified AdmSCr) to ascertain baseline SCr [14, 15].

Consensus definitions of AKI and baseline SCr are critical for facilitating comparisons of clinical studies of pediatric AKI. This study assessed AKI incidence and its association with mortality occurring during the stay of pediatric intensive care unit (PICU) under different pediatric AKI and baseline SCr definitions to determine the preferable approach for diagnosing pediatric AKI.

## Methods

### Study population

This multicenter prospective cohort study was conducted in the PICUs of four tertiary hospitals (Children’s Hospital of Soochow University, Children’s Hospital of Fudan University, Anhui Provincial Children’s Hospital, and Xuzhou Children’s Hospital) from September 2020 to February 2021. Children who were aged 1 month to 18 years and met the criteria for PICU admission [16] were eligible for enrollment. Children with chronic kidney disease (CKD) stage 3 and above or those who underwent renal replacement therapy (RRT) before admission were excluded. The study was approved by the Institutional Review Board/Ethical Committee of the hospitals and performed in accordance with the Declaration of Helsinki, and parental written informed consent was obtained from all participants.

### Clinical data collection

Baseline patient characteristics were recorded within the first 24 h after PICU admission, including patients’ demographics, medical history, SCr values obtained in the 3 months before PICU admission and the main reason for admission. Clinical status, comorbidities, and therapeutic intervention and medication were recorded until PICU discharge or death. The severity of illness was determined using the pediatric risk of mortality III (PRISM III) score as described in our previous studies [14, 15].

### Definition of AKI and estimation method of baseline SCr

The diagnosis and staging of AKI were determined using the following criteria: KDIGO criterion with SCr [7]: SCr rise ≥ 0.3 mg/dL within 48 h or ≥ 1.5 times the baseline SCr within 7 days. Stage 1, SCr rise ≥ 0.3 mg/dL within 48 h, or ≥ 1.5–1.9 times the baseline SCr within 7 days; stage 2, SCr ≥ 2.0–2.9 times the baseline SCr within 7 days; stage 3, SCr ≥ 3.0 times the baseline SCr or ≥ 4.0 mg/dL within 7 days, or initiation of RRT, or in patients < 18 years, decrease in estimated GFR < 35 mL/min/1.73 m^2^. Modified KDIGO: the SCr increase should reach a concentration of at least 0.5 mg/dL when the KDIGO criterion with SCr is applied to define AKI and AKI stage [17]. The pROCK definition [10]: SCr rise ≥ 0.2 mg/dL and ≥ 1.3 times the baseline SCr within 7 days. Stage 1, SCr rise ≥ 0.2–0.4 mg/dL and ≥ 1.3–1.59 times the baseline SCr; stage 2, SCr rise ≥ 0.5–0.9 mg/dL and ≥ 1.6–2.19 times the baseline SCr; stage 3, SCr rise ≥ 1.0 mg/dL and ≥ 2.2 times the baseline SCr. AKI stage 1 was defined as mild AKI, and AKI stage 2 or 3 was defined as severe AKI [10].

The following four methods were used to estimate the baseline SCr. Schwartz method: Back calculation with the bedside Schwartz formula (*k* × height (cm)/serum creatinine (mg/dL), *k* = 0.413) assuming a GFR of 120 mL/min/1.73 m^2^ as previously described and validated [18]. NormsMax method: Maximum published normative SCr values for age and sex [19]. AdmSCr method: SCr level at the time of PICU admission [13]. Modified AdmSCr method: Same as the AdmSCr method except for patients with elevated SCr ≥ 1.2 mg/dL at admission, for whom the lowest SCr value within 2 weeks during the PICU stay was considered the baseline SCr [14, 15].

Blood samples for SCr measurement were collected and tested on PICU admission, followed by routine measurement every 48–72 h during the first week and every 3–5 days until death or discharge from the PICU. The sarcosine oxidase method was used for SCr measurement.

### Clinical outcome

The outcome of interest was PICU mortality, defined as death during the PICU stay, including death due to withdrawal of treatment.

### Statistical analysis

Statistical analyses were performed with the software of SPSS and Medcalc. The chi-square test or Fisher’s exact test was applied for comparisons of categorical variables. The concordance rate and weighted kappa (*κ*) value of the kappa statistic were used to evaluate the consistency of AKI or AKI stage according to different definitions based on different baseline SCrs. Univariate and multivariate logistic regression analyses were used to calculate the odds ratio (OR) and adjusted OR to assess the association of AKI and AKI stage with mortality, and the area under the receiver operating characteristic curve (AUC) was calculated to assess the discriminative power. *P* value < 0.05 (two-tailed) was considered statistically significant.

## Results

### Patient characteristics

**Fig. 1:**
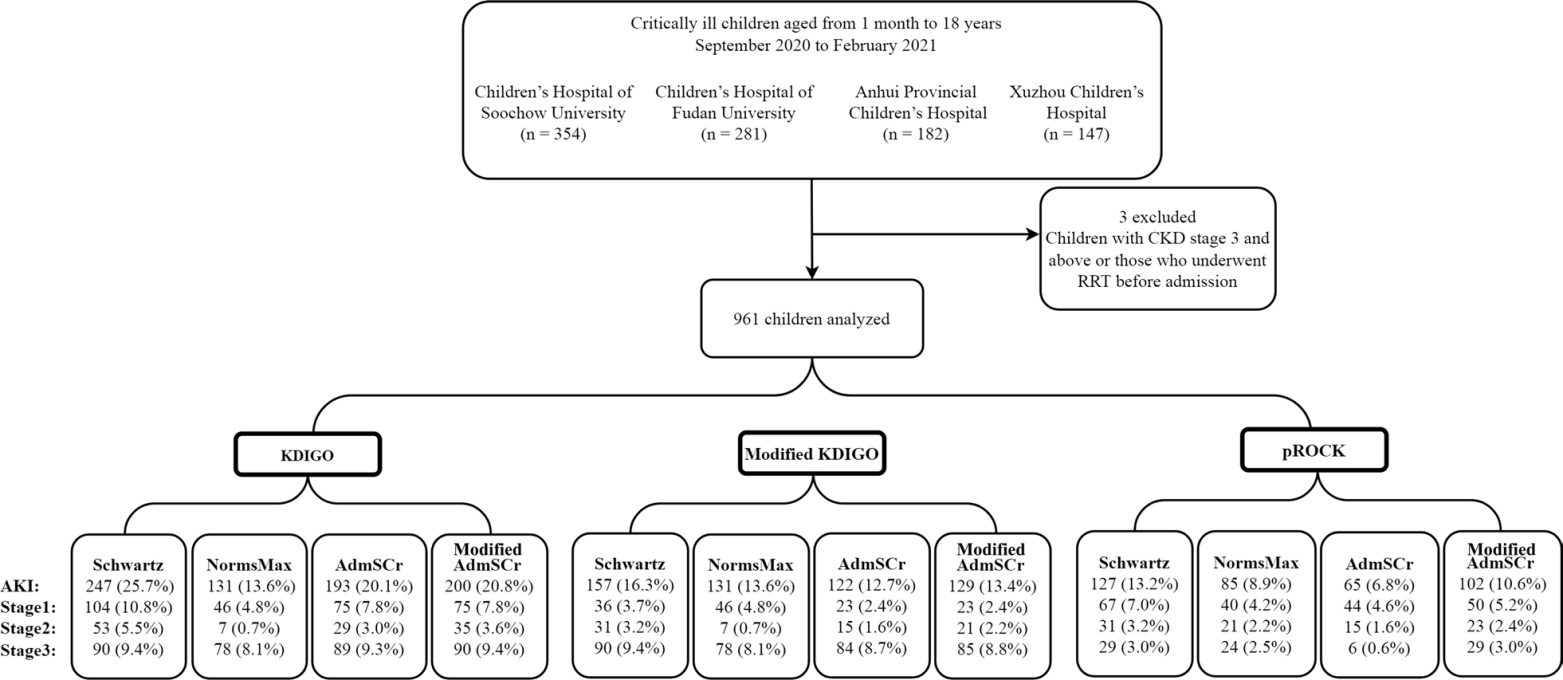
Diagram of cases included in the study and AKI incidence. AKI was defined and staged according to the KDIGO, modified KDIGO, and pROCK definitions. The baseline SCr was calculated based on the Schwartz formula or estimated as the NormsMax, AdmSCr and modified AdmSCr methods. *AdmSCr* admission serum creatinine, *KDIGO* Kidney Disease: Improving Global Outcome, *NormsMax* the upper normative value, *pROCK* pediatric reference change value optimized for acute kidney injury

The study involved 961 patients in the final analysis. Of the 964 patients who met the criteria for PICU admission, 3 with CKD stage 5 were excluded. Details are shown in Fig. 1. The demographic and clinical characteristics of the patients are described in Additional file 1: Table S1.

### AKI incidence

**Fig. 2:**
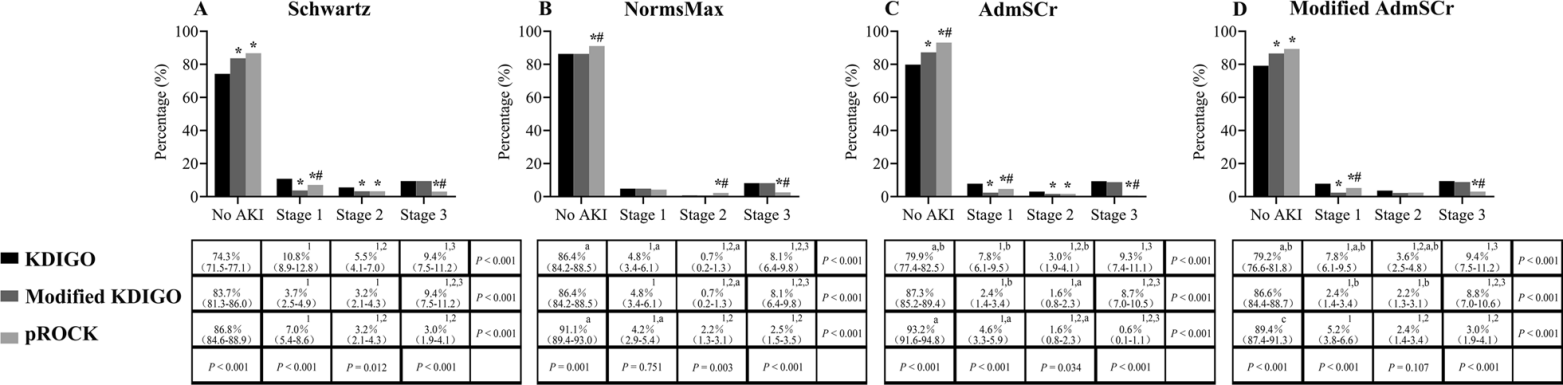
Incidence and stages of AKI under different AKI definitions and baseline SCr estimation methods. Incidence rates are presented as percentages with 95% confidence intervals. *P < 0.05 versus KDIGO; ^#^*P* < 0.05 versus modified KDIGO; ^1^*P* < 0.05 versus No AKI; ^2^*P* < 0.05 versus AKI stage 1; ^3^*P* < 0.05 versus AKI stage 2; ^a^*P* < 0.05 versus Schwartz; ^b^*P* < 0.05 versus NormsMax; ^c^*P* < 0.05 versus AdmSCr

The AKI incidence and severity distribution are shown in Fig. 2, Additional file 2: Figure S1 and Additional file 3: Figure S2. The proportion of patients with AKI by KDIGO was higher than that using the other two definitions based on any baseline SCr estimation method (Fig. 2A, C, D), except for the NormsMax method. When using the NormMax method, the KDIGO and modified KDIGO definitions led to an identical AKI incidence (Fig. 2B).

For the KDIGO criterion, AKI proportion based on the Schwartz method was higher than that based on the other three baseline SCr estimation methods. For the modified KDIGO definition, AKI proportion based on the Schwartz method was similar to that based on the other three baseline SCr estimation methods. For the pROCK definition, AKI proportion based on the Schwartz method was higher than that based on NormsMax and AdmSCr.

### Interdefinition agreement

As shown in Additional file 1: Tables S2 and S3, the concordance rates of AKI stages between the KDIGO and modified KDIGO definitions based on any baseline SCr estimation method were > 90.0% with *κ* values > 0.8, and an agreement of 100% was achieved when using NormsMax. In addition, the concordance rates of AKI stages between different baseline SCr estimation methods were all > 90.0% with all *κ* values > 0.8 when using the modified KDIGO definition. The AdmSCr and modified AdmSCr methods achieved the most similar distribution of AKI severity strata, whichever AKI definition was used.

### AKI, AKI stage and mortality

**Fig. 3:**
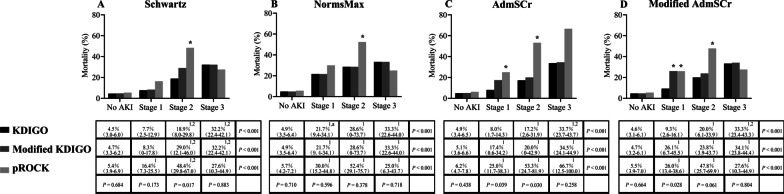
PICU mortality under different AKI definitions and baseline SCr estimation methods. Mortality rates are presented as percentages with 95% confidence intervals. *P < 0.05 versus KDIGO; ^1^*P* < 0.05 versus No AKI; ^2^*P* < 0.05 versus AKI stage 1; ^a^*P* < 0.05 versus Schwartz

The overall PICU mortality rate was 8.2%. Mortality was significantly higher in AKI than in non-AKI patients across all definitions based on any baseline SCr estimation method, as shown in Fig. 3 and Additional file 4: Figure S3.

The mortality rate of AKI was lowest with the KDIGO definition, except when NormsMax was used, for which the mortality in each stage of AKI according to the KDIGO and modified KDIGO definitions was exactly the same (Fig. 3 and Additional file 5: Figure S4). For the KDIGO criterion, the mortality rate was lower in AKI patients based on the Schwartz method than in those based on NormsMax (Fig. 3 and Additional file 5: Figure S4). For the modified KDIGO and pROCK definitions, the mortality rates in AKI patients did not differ between different baseline SCr estimation methods, nor was mortality in each AKI stage (Fig. 3 and Additional file 5: Figure S4).

Additionally, the mortality rate of patients who had KDIGO-defined AKI with an increase in SCr to < 0.5 mg/dL was similar to those without AKI (Schwartz, 6.7% vs. 4.5%, *P* = 0.42; AdmSCr, 7.0% vs. 4.9%, *P* = 0.40; modified AdmSCr, 4.3% vs. 4.6%, *P* = 0.92).

As regarding to AKI staging, the differences in PICU mortality encountered when comparing AKI definitions are shown in Fig. 3 and Additional file 4: Figure S3; and were further evaluated among patients with non-AKI, mild AKI and severe AKI in Additional file 1: Table S4. For the KDIGO criterion, PICU mortality increased pursuant to staging, and higher PICU mortality was observed in children with severe AKI compared to those with mild AKI, except that the mortality in each stage of AKI based on the NormsMax method did not differ (Fig. 3 and Additional file 1: Table S4). For the modified KDIGO definition, only based on the Schwartz method, children with severe AKI had higher PICU mortality than children with mild AKI (Additional file 1: Table S4). For the pROCK definition, PICU mortality did not increase pursuant to staging (Fig. 3).

### Performance of different AKI and baseline SCr definitions for PICU mortality

**Fig. 4:**
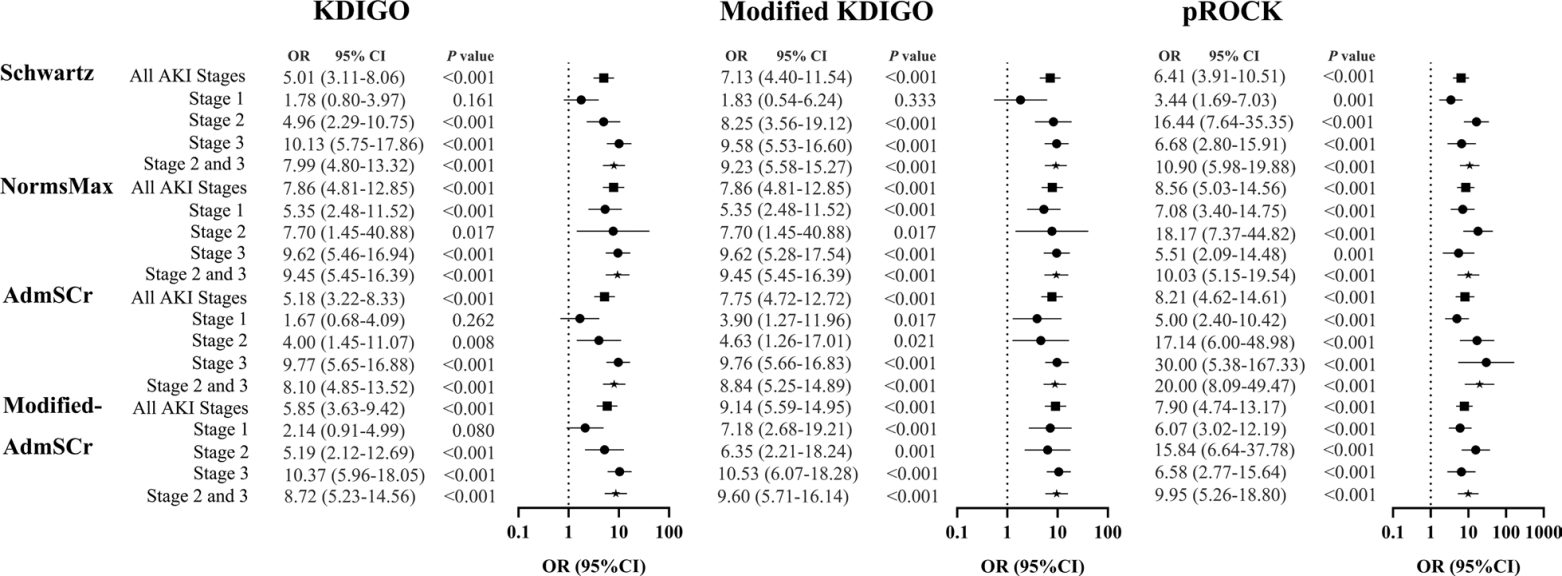
Odds ratios for death in patients with and without AKI according to different criteria. Univariate logistic regression analysis with non-AKI as reference

AKI was associated with increased PICU mortality in univariate and multivariate logistic regression analyses (Fig. 4 and Additional file 6: Figure S5). In examining AKI by stage, more severe AKI stages defined by KDIGO or modified KDIGO had higher unadjusted odds for mortality (Fig. 4). AKI stage 3 defined by KDIGO or modified KDIGO remained associated with increased PICU mortality after adjusting for confounders, including age, sex and illness severity assessed by the PRISM III score, but the association was not observed when using pROCK (Additional file 6: Figure S5).

**Fig. 5:**
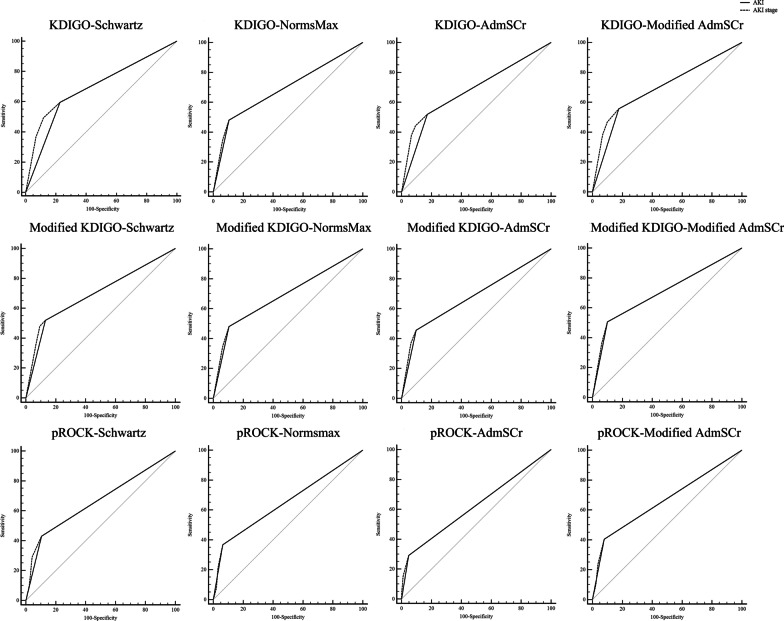
Predictive performance of AKI and AKI stage for mortality. AKI was defined by the KDIGO, modified KDIGO and pROCK definitions based on the Schwartz, NormsMax, AdmSCr and modified AdmSCr estimation methods, respectively

The predictive ability of AKI and AKI stage assessed with the AUC is shown in Fig. 5 and Additional file 1: Table S5. Both AKI and AKI stage defined by the modified KDIGO based on the modified AdmSCr method displayed AUCs above 0.7. Both AKI and AKI stage according to the pROCK definition displayed the lowest AUCs, whichever baseline SCr estimation method used.

## Discussion

This multicenter prospective cohort study demonstrates that the incidence and staging of AKI vary depending on the AKI definition and the baseline SCr used. Critically ill children with AKI diagnosis, regardless of the definition of AKI and the baseline SCr used, have higher PICU mortality.

Since no consensus exists regarding which AKI definition to use in children, Sutherland et al. compared AKI incidence and associated outcomes generated using the pRIFLE, AKIN, and KDIGO criteria in children and indicates that the KDIGO definition offers applicability to pediatric population and highlights the necessity of a unified AKI definition for children [2]. The diagnosis of AKI is reliance on a creatinine-based definition. Low and varying levels of SCr are characteristic in young children [10], and a small increase in SCr, even adjusted for age and baseline SCr, may meet the AKI criterion. To prevent a bias toward classifying AKI as present in young infants, Selewski et al. modified the KDIGO criterion by stipulating that the SCr should reach a concentration of at least 0.5 mg/dL for defining pediatric AKI [9]. The study showed much lower AKI incidence in PICU population [9] compared with that obtained in a pediatric study using the KDIGO definition [2]. Our results are consistent with the previous reports [2, 9], and we also noted that the largest disparity of AKI incidence was in stage 1, indicating that the KDIGO definition is more sensitive, identifying a great number of mild AKI, than modified KDIGO in the diagnosis of pediatric AKI.

In addition, the pROCK, an AKI definition based on reference change value of SCr adjusted for age and baseline SCr in children, was proposed, in which a large proportion of children with AKI stage 1 defined by KDIGO were classified as non-AKI according to pROCK [10]. The investigators conclude that the pROCK should be used as AKI criterion for children because that using pROCK may avoid overdiagnosis of AKI [10]. However, some scholars have questioned the proposal. An editorial by Goldstein suggests that the SCr level does not rise until renal reserve has been removed, and an overemphasis on optimizing the specificity of diagnostic criteria might lead to clinicians not ordering follow-up tests for patients with the risk of AKI progression [20]. The largest disparity of AKI incidence is in stage 1, which, however, often progresses to more severe stages. Using overly specific criteria, such as pROCK, might miss the brief window to initiate any potential intervention and induce additional negative consequences [20]. In our study, when using the pROCK definition, the incidence of both mild and severe AKI was much lower, PICU mortality did not increase pursuant to staging, AKI stage 3 was not associated with PICU mortality after adjustment for confounders, and pROCK had the lowest mortality predictability, which were all contrary to previous studies [10, 21, 22]. We speculate that these might be because that the pROCK definition does not consider patients receiving RRT or with estimated GFR < 35 mL/min/1.73 m^2^ as AKI stage 3.

The editorial by Goldstein also indicates that the more specific criteria may be useful for studying AKI-related outcomes [20]. In our study, the PICU mortality rate of AKI with an increase in SCr to ≤ 0.5 mg/dL resembled that without KDIGO-defined AKI, which is consistent with the report by Holmes et al. [23], and the modified KDIGO-defined AKI was more strongly associated with related mortality than KDIGO-defined AKI, suggesting that modified KDIGO may have a higher probability of accurately predicting the risk of mortality. Furthermore, considering the strong consistencies of AKI stages between different baseline SCr estimation methods when using modified KDIGO, we prefer the modified KDIGO definition.

Many patients have no baseline SCr available [24]. The methods for estimating baseline SCr differ remarkably in different studies, in turn leading to substantial differences in AKI incidence and AKI severity description [25–28]. As a tool for estimating baseline SCr in children, the back-calculation method with the Schwartz formula has become widespread [11], but some scholars questioned this method. A pediatric study showed that the baseline SCr obtained based on the Schwartz formula was lower than the actual baseline level, and the researchers conclude that the Schwartz method would lead to overdiagnosis of AKI in children [23]. Back-calculation relies only on age, height and sex and does not take into account the actual renal function of the patient, which may have partly caused this result. Indeed, when using the KDIGO definition, the Schwartz method led to the highest AKI incidence, but the lowest associated mortality among all baseline SCr estimation methods in our study. However, this disparity was not observed when using the modified KDIGO definition, suggesting that the disparity may be overcome by using the modified KDIGO.

The sex- and age-based reference intervals might also be the preferable approach for generating baseline SCr in the absence of the actual baseline SCr [23, 29]. However, to date, there is no consensus on what reference intervals to use. Our study accepted the sex- and age-specific reference intervals from healthy children of the wider community [19] because they were derived from a large collaborative study among pediatric centers that addresses critical gaps in pediatric reference intervals [19]. When using the upper normative value (NormsMax) as the baseline SCr, the KDIGO and modified KDIGO demonstrated 100% agreement in distribution of AKI severity strata. However, PICU mortality did not increase in accordance with AKI severity using NormsMax, which is consistent with a pediatric study [23]. Therefore, sex- and age-specific reference intervals may not be the best choice for generating baseline SCr.

Using the first available SCr value following hospital admission as baseline SCr is also an estimation method, but it may be misleading in patients with community-acquired AKI [30]. To prevent the missed diagnosis of AKI in critically ill children, our research team modified the AdmSCr method that had always been adopted in our previous studies [14, 15]. For the same AKI definition, the modified AdmSCr had slightly greater mortality predictability. However, whether the small difference hold significant clinical value requires further validation. Siew et al. reported that the AdmSCr method underestimates AKI incidence in adults, which was not observed in our study [31]. This discrepancy might be due to adults being more likely to have missed diagnosis of community-acquired AKI. Additionally, PICU mortality increased pursuant to staging based on the AdmSCr or modified AdmSCr method according to the KDIGO definition. The above results suggest that the AdmSCr and modified AdmSCr methods may be chosen to define baseline SCr when using the KDIGO definition to diagnose AKI in children.

The study has some limitations. First, since we were interested in the impact of baseline SCr and the pROCK definition does not include urine output, only SCr was applied to define pediatric AKI in all definitions. Second, we did not compare the estimated with the actual baseline SCr, which was lacking for most children. Third, we cannot generalize our findings to patients outside the pediatric ICU setting.

## Conclusions

Different AKI definitions and baseline SCr estimation methods lead to differences in AKI incidence and staging. Both KDIGO and modified KDIGO definitions offer advantages. The KDIGO definition may be sensitive, identifying a great number of mild AKI. The modified KDIGO may be the preferable approach, defining AKI which is highly relevant to PICU mortality in critically ill children. Based on the Schwartz baseline SCr estimation method, the modified KDIGO may provide promise for improving clinicians’ ability to diagnose AKI.

## Supplementary Information

**Additional file 1: Table S1.** Demographic and clinical characteristics of patients. **Table S2.** Concordance of AKI designation according to different AKI definitions. **Table S3.** Concordance of AKI designation based on different baseline SCr estimation methods. **Table S4.** Death risk stratified by the status and severity of AKI. **Table S5.** Predictive performance of AKI and AKI stages for mortality.

**Additional file 2: Figure S1.** Incidence and stages of AKI based on different baseline SCr estimation methods. Values are presented as percentages with 95% confidence intervals. ^1^*P* < 0.05 vs. No AKI; ^2^*P* < 0.05 vs. AKI stage 1; ^3^*P* < 0.05 vs. AKI stage 2; ^a^*P* < 0.05 vs. Schwartz; ^b^*P* < 0.05 vs. NormsMax; ^c^*P* < 0.05 vs. AdmSCr.

**Additional file 3: Figure S2.** AKI stages under different AKI definitions and baseline SCr estimation methods. ^*^P < 0.05 vs. KDIGO; ^#^*P* < 0.05 vs. Modified KDIGO; ^a^*P* < 0.05 vs. Schwartz; ^b^*P* < 0.05 vs. NormsMax; ^c^*P* < 0.05 vs. AdmSCr.

**Additional file 4: Figure S3.** PICU mortality by AKI stage based on different baseline SCr estimation methods. Values are presented as percentages with 95% confidence intervals. ^1^*P* < 0.05 vs. No AKI; ^2^*P* < 0.05 vs. AKI stage 1;.^a^*P* < 0.05 vs. Schwartz.

**Additional file 5: Figure S4.** Mortality rate in patients with AKI. ^*^P < 0.05 vs. KDIGO; ^a^*P* < 0.05 vs. Schwartz.

**Additional file 6: Figure S5.** Adjusted odds ratios for death in patients with and without AKI by different criteria. Multivariate logistic regression analysis with non-AKI as the reference; confounders included in the analysis were age, sex and pediatric risk of mortality III score.

## Data Availability

The datasets used and/or analyzed during the current study are available from the corresponding author on reasonable request.

## Abbreviations

AdmSCr: Admission serum creatinine
AKI: Acute kidney injury
AUC: Area under the receiver operating characteristic curve
CKD: Chronic kidney disease
GFR: Glomerular filtration rate
KDIGO: Kidney Disease: Improving Global Outcome
NormsMax: The upper normative value
OR: Odds ratio
PICU: Pediatric intensive care unit
pROCK: Pediatric reference change value optimized for acute kidney injury
RRT: Renal replacement therapy
SCr: Serum creatinine
