# Supplementary material for: Comparison of diagnostic criteria for acute kidney injury in critically ill children: a multicenter cohort study
Source: Crit Care. 2022 Jul 7;26:207. doi: 10.1186/s13054-022-04083-0 (PMC9264539; doi:10.1186/s13054-022-04083-0)
Supplement: Supplementary file 1 — Additional file 1: Table S1. Demographic and clinical characteristics of patients. Table S2. Concordance of AKI designation according to different AKI definitions. Table S3. Concordance of AKI designation based on different baseline SCr estimation methods. Table S4. Death risk stratified by the status and severity of AKI. Table S5. Predictive performance of AKI and AKI stages for mortality. [file 13054_2022_4083_MOESM1_ESM.doc]

Table S1. Demographic and clinical characteristics of patients

|  | All patients (n = 961) |
| --- | --- |
| Age, months | 32.0 [9.5-84.5] |
| Weight, kg | 13.0 [8.0-24.0] |
| Male, n | 568 (59.1) |
| Admission diagnosis |  |
| Respiratory diseases, n | 230 (23.9) |
| Neurological diseases, n | 249 (25.9) |
| Digestive diseases, n | 91 (9.5) |
| Hematologic diseases, n | 100 (10.4) |
| Sepsis, n | 118 (12.3) |
| Others, n | 173 (18.0) |
| Admission SCr, mg/dL | 0.3 [0.2-0.4] |
| MV, n | 276 (28.7) |
| RRT, n | 55 (5.7) |
| PRISM III score | 4 [2-9] |
| Hospital stay, days | 12 [7-21] |
| PICU stay, days | 5 [2-9] |
| Death, n | 79 (8.2) |

*MV* mechanical ventilation, *PICU* pediatric intensive care unit, *PRISM III* pediatric risk of mortality III, *RRT* renal replacement therapy, *SCr* serum creatinine

Table S2. Concordance of AKI designation according to different AKI definitions

|  |  | KDIGO |  |  |  | Modified KDIGO | | |
| --- | --- | --- | --- | --- | --- | --- | --- | --- |
|  |  | Weightd kappa | Concordance (%) | P value |  | Weighted kappa | Concordance (%) | P value |
| Schwartz | Modified KDIGO | 0.843 | 90.6 | < 0.001 |  |  |  |  |
|  | pROCK | 0.542 | 79.9 | < 0.001 |  | 0.647 | 88.3 | < 0.001 |
| NormsMax | Modified KDIGO | 1.0 | 100 | < 0.001 |  |  |  |  |
|  | pROCK | 0.608 | 91.3 | < 0.001 |  | 0.608 | 91.3 | < 0.001 |
| AdmSCr | Modified KDIGO | 0.846 | 92.6 | < 0.001 |  |  |  |  |
|  | pROCK | 0.312 | 81.9 | < 0.001 |  | 0.368 | 88.3 | < 0.001 |
| Modified  AdmSCr | Modified KDIGO | 0.851 | 92.6 | < 0.001 |  |  |  |  |
|  | pROCK | 0.538 | 83.7 | < 0.001 |  | 0.632 | 90.1 | < 0.001 |

*AdmSCr* admission serum creatinine, *KDIGO* Kidney Disease Improving Global Outcomes, *NormsMax* upper normative values, *pROCK* pediatric reference change value optimized for acute kidney injury

Table S3. Concordance of AKI designation based on different baseline SCr estimation methods

|  |  | Schwartz |  |  |  | NormsMax |  |  |  | AdmSCr | | |
| --- | --- | --- | --- | --- | --- | --- | --- | --- | --- | --- | --- | --- |
|  |  | Weighted kappa | Concordance (%) | *P* value |  | Weighted kappa | Concordance (%) | *P* value |  | Weightd kappa | Concordance (%) | *P* value |
| KDIGO | NormsMax | 0.721 | 84.3 | < 0.001 |  |  |  |  |  |  |  |  |
|  | AdmSCr | 0.629 | 78.6 | < 0.001 |  | 0.749 | 87.8 | < 0.001 |  |  |  |  |
|  | Modified AdmSCr | 0.657 | 79.3 | < 0.001 |  | 0.759 | 88.0 | < 0.001 |  | 0.969 | 98.4 | < 0.001 |
| Modified KDIGO | NormsMax | 0.873 | 93.7 | < 0.001 |  |  |  |  |  |  |  |  |
|  | AdmSCr | 0.833 | 92.8 | < 0.001 |  | 0.893 | 95.2 | < 0.001 |  |  |  |  |
|  | Modified AdmSCr | 0.864 | 93.5 | < 0.001 |  | 0.901 | 95.4 | < 0.001 |  | 0.966 | 98.6 | < 0.001 |
| pROCK | NormsMax | 0.818 | 93.7 | < 0.001 |  |  |  |  |  |  |  |  |
|  | AdmSCr | 0.416 | 88.4 | < 0.001 |  | 0.414 | 90.7 | < 0.001 |  |  |  |  |
|  | Modified AdmSCr | 0.783 | 92.2 | < 0.001 |  | 0.798 | 93.8 | < 0.001 |  | 0.648 | 95.8 | < 0.001 |

*AdmSCr* admission serum creatinine, *KDIGO* Kidney Disease Improving Global Outcomes, *NormsMax* upper normative values, *pROCK* pediatric reference change value optimized for acute kidney injury

Table S4. Death risk stratified by the status and severity of AKI

|  |  | No AKI | |  | Mild AKI | |  | Severe AKI | |
| --- | --- | --- | --- | --- | --- | --- | --- | --- | --- |
|  |  | Total  n (%) | Death  n (%) |  | Total  n (%) | Death  n (%) |  | Total  n (%) | Death  n (%) |
| Schwartz | KDIGO | 714 (74.3) | 32 (4.5) |  | 104 (10.8) | 8 (7.7) |  | 143 (14.9) | 39 (27.3)^1, 2^ |
|  | Modified KDIGO | 804 (83.7)^a^ | 38 (4.7) |  | 36 (3.7)^a^ | 3 (8.3) |  | 121 (12.6) | 38 (31.4) ^1, 2^ |
|  | pROCK | 834 (86.8)^a^ | 45 (5.4) |  | 67 (7.0)^a, b^ | 11 (16.4)^1^ |  | 60 (6.2)^a, b^ | 23 (38.3) ^1, 2^ |
|  | *P* value | < 0.001 | 0.68 |  | < 0.001 | 0.17 |  | < 0.001 | 0.29 |
| NormsMax | KDIGO | 830 (86.4) | 41 (4.9) |  | 46 (4.8) | 10 (21.7)^1^ |  | 85 (8.8) | 28 (32.9)^1^ |
|  | Modified KDIGO | 830 (86.4) | 41 (4.9) |  | 46 (4.8) | 10 (21.7)^1^ |  | 85 (8.8) | 28 (32.9)^1^ |
|  | pROCK | 876 (91.1)^a, b^ | 50 (5.7) |  | 40 (4.2) | 12 (30.0)^1^ |  | 45 (4.7)^a, b^ | 17 (37.8)^1^ |
|  | *P* value | 0.001 | 0.71 |  | 0.75 | 0.60 |  | < 0.001 | 0.56 |
| AdmSCr | KDIGO | 768 (79.9) | 38 (4.9) |  | 75 (7.8) | 6 (8.0) |  | 118 (12.3) | 35 (29.7) ^1, 2^ |
|  | Modified KDIGO | 839 (87.3)^a^ | 43 (5.1) |  | 23 (2.4)^a^ | 4 (17.4)^1^ |  | 99 (10.3) | 32 (32.3)^1^ |
|  | pROCK | 896 (93.2)^a, b^ | 56 (6.2) |  | 44 (4.6)^a, b^ | 11 (25.0)^a, 1^ |  | 21 (2.2)^a, b^ | 12 (57.1)^a, b, 1, 2^ |
|  | *P* value | < 0.001 | 0.44 |  | < 0.001 | 0.04 |  | < 0.001 | 0.05 |
| Modified AdmSCr | KDIGO | 761 (79.2) | 35 (4.6) |  | 75 (7.8) | 7 (9.3) |  | 125 (13.0) | 37 (29.6) ^1, 2^ |
|  | Modified KDIGO | 832 (86.6)^a^ | 39 (4.7) |  | 23 (2.4)^a^ | 6 (26.1)^a, 1^ |  | 106 (11.0) | 34 (32.1)^1^ |
|  | pROCK | 859 (89.4)^a^ | 47 (5.5) |  | 50 (5.2)^a, b^ | 13 (26.0)^a, 1^ |  | 52 (5.4)^a, b^ | 19 (36.5)^1^ |
|  | *P* value | < 0.001 | 0.66 |  | < 0.001 | 0.03 |  | < 0.001 | 0.66 |

AKI stage 1 was defined as mild AKI, and AKI stage 2 or 3 was defined as severe AKI.

^a^*P* <0.05 vs. KDIGO

^b^*P* <0.05 vs. Modified KDIGO

^1^*P* <0.05 vs. No AKI

^2^*P* <0.05 vs. Mild AKI

*AKI* acute kidney injury, *AdmSCr* admission serum creatinine*, KDIGO* Kidney Disease Improving Global Outcomes, *NormsMax* upper normative values, *pROCK* pediatric reference change value optimized for acute kidney injury

Table S5. Predictive performance of AKI and AKI stages for mortality

|  |  | Schwartz | |  | NormsMax | |  | AdmSCr | |  | Modified AdmSCr | |
| --- | --- | --- | --- | --- | --- | --- | --- | --- | --- | --- | --- | --- |
|  |  | AKI | AKI stages |  | AKI | AKI stages |  | AKI | AKI stages |  | AKI | AKI stages |
| KDIGO | AUC | 0.68 | 0.71 |  | 0.69 | 0.69 |  | 0.67 | 0.69 |  | 0.69 | 0.71 |
|  | 95% CI | 0.65-0.71 | 0.6-0.74 |  | 0.66-0.72 | 0.66-0.72 |  | 0.64-0.70 | 0.66-0.72 |  | 0.66-0.72 | 0.68-0.74 |
|  | *P* value | < 0.001 | < 0.001 |  | < 0.001 | < 0.001 |  | < 0.001 | < 0.001 |  | < 0.001 | < 0.001 |
|  | Sensitivity, % | 60 | 49 |  | 48 | 48 |  | 52 | 44 |  | 56 | 56 |
|  | 95% CI | 48-70 | 38-61 |  | 37-60 | 37-60 |  | 40-63 | 33-56 |  | 44-67 | 44-67 |
|  | Specificity, % | 77 | 88 |  | 90 | 90 |  | 83 | 91 |  | 82 | 82 |
|  | 95% CI | 74-80 | 86-90 |  | 87-91 | 87-91 |  | 80-85 | 89-92 |  | 80-85 | 80-85 |
|  | PPV, % | 19 | 27 |  | 29 | 29 |  | 21 | 30 |  | 22 | 22 |
|  | 95% CI | 16-23 | 22-33 |  | 23-36 | 23-36 |  | 17-26 | 23-37 |  | 18-26 | 18-26 |
|  | NPV , % | 96 | 95 |  | 95 | 95 |  | 95 | 95 |  | 95 | 95 |
|  | 95% CI | 94-97 | 94-96 |  | 94-96 | 94-96 |  | 94-96 | 94-96 |  | 94-96 | 94-96 |
| Modified  KDIGO | AUC  95% CI | 0.69  0.66-0.72 | 0.70  0.67-0.73 |  | 0.69  0.66-0.72 | 0.69  0.66-0.72 |  | 0.68  0.65-0.71 | 0.68 0.65-0.71 |  | 0.70  0.67-0.73 | 0.71  0.68-0.73 |
|  | *P* value | < 0.001 | < 0.001 |  | < 0.001 | < 0.001 |  | < 0.001 | < 0.001 |  | < 0.001 | < 0.001 |
|  | Sensitivity, % | 52 | 52 |  | 48 | 48 |  | 46 | 46 |  | 51 | 51 |
|  | 95% CI | 40-63 | 40-63 |  | 37-60 | 37-60 |  | 34-57 | 34-57 |  | 39-62 | 39-62 |
|  | Specificity, % | 87 | 87 |  | 90 | 90 |  | 90 | 90 |  | 90 | 90 |
|  | 95% CI | 84-89 | 84-89 |  | 87-91 | 87-91 |  | 88-92 | 88-92 |  | 88-92 | 88-92 |
|  | PPV, % | 26 | 26 |  | 29 | 29 |  | 30 | 30 |  | 31 | 31 |
|  | 95% CI | 21-32 | 21-32 |  | 23-36 | 23-36 |  | 23-36 | 23-36 |  | 25-38 | 25-38 |
|  | NPV, % | 95 | 95 |  | 95 | 95 |  | 95 | 95 |  | 95 | 95 |
|  | 95% CI | 94-96 | 94-96 |  | 94-96 | 94-96 |  | 94-96 | 94-96 |  | 94-96 | 94-96 |
| pROCK | AUC | 0.66 | 0.67 |  | 0.65 | 0.65 |  | 0.62 | 0.62 |  | 0.66 | 0.66 |
|  | 95% CI | 0.63-0.69 | 0.64-0.70 |  | 0.62-0.68 | 0.62-0.68 |  | 0.59-0.65 | 0.59-0.66 |  | 0.63-0.69 | 0.63-0.69 |
|  | *P* value | < 0.001 | < 0.001 |  | < 0.001 | < 0.001 |  | < 0.001 | < 0.001 |  | < 0.001 | < 0.001 |
|  | Sensitivity, % | 43 | 43 |  | 37 | 37 |  | 29 | 29 |  | 41 | 41 |
|  | 95% CI | 32-55 | 32-55 |  | 26-48 | 26-48 |  | 19-40 | 19-40 |  | 30-52 | 30-52 |
|  | Specificity, % | 90 | 90 |  | 94 | 94 |  | 95 | 95 |  | 92 | 92 |
|  | 95% CI | 87-91 | 87-91 |  | 92-95 | 92-95 |  | 94-97 | 94-97 |  | 90-94 | 90-94 |
|  | PPV, % | 27 | 27 |  | 34 | 34 |  | 35 | 35 |  | 31 | 31 |
|  | 95% CI | 21-33 | 21-33 |  | 26-43 | 26-43 |  | 26-46 | 26-46 |  | 24-39 | 24-39 |
|  | NPV, % | 95 | 95 |  | 94 | 94 |  | 94 | 94 |  | 95 | 95 |
|  | 95% CI | 94-96 | 94-96 |  | 93-95 | 93-95 |  | 93-95 | 93-95 |  | 94-96 | 94-96 |

*AKI* acute kidney injury, *AdmSCr* admission serum creatinine, *AUC* area under the receiver operating characteristic curve*, KDIGO* Kidney Disease Improving Global Outcomes, *NormsMax* upper normative values, *NPV* negative predictive value, *PPV* positive predictive value, *pROCK* pediatric reference change value optimized for acute kidney injury
