# Supplementary material for: Comparison of diagnostic criteria for acute kidney injury in critically ill children: a multicenter cohort study
Source: Crit Care. 2022 Jul 7;26:207. doi: 10.1186/s13054-022-04083-0 (PMC9264539; doi:10.1186/s13054-022-04083-0)
Supplement: Supplementary file 2 [file 13054_2022_4083_MOESM2_ESM.docx]

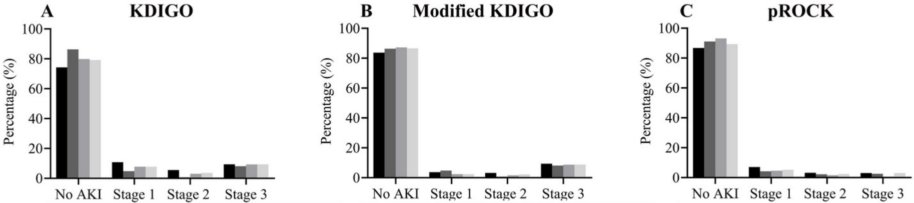

A KDIGO

	No AKI	Stage 1	Stage 2	Stage 3	
Schwartz	74.3% (71.5-77.1)	10.8%[1] (8.9-12.8)	5.5%[1,2] (4.1-7.0)	9.4%[1,3] (7.5-11.2)	$P < 0.001$
Normsmax	86.4%[a] (84.2-88.5)	4.8%[1,a] (3.4-6.1)	0.7%[1,2,a] (0.2-1.3)	8.1%[1,2,3] (6.4-9.8)	$P < 0.001$
AdmSCr	79.9%[a,b] (77.4-82.5)	7.8%[1,a,b] (6.1-9.5)	3.0%[1,2,a,b] (1.9-4.1)	9.3%[1,3] (7.4-11.1)	$P < 0.001$
Modified AdmSCr	79.2%[a,b] (76.6-81.8)	7.8%[1,a,b] (6.1-9.5)	3.6%[1,2,a,b] (2.5-4.8)	9.4%[1,3] (7.5-11.2)	$P < 0.001$
	$P < 0.001$	$P < 0.001$	$P < 0.001$	$P = 0.729$	

B Modified KDIGO

	No AKI	Stage 1	Stage 2	Stage 3	
Schwartz	83.7% (81.3-86.0)	3.7%[1] (2.5-4.9)	3.2%[1] (2.1-4.3)	9.4%[1,2,3] (7.5-11.2)	$P < 0.001$
Normsmax	86.4% (84.2-88.5)	4.8%[1] (3.4-6.1)	0.7%[1,2,a] (0.2-1.3)	8.1%[1,2,3] (6.4-9.8)	$P < 0.001$
AdmSCr	87.3% (85.2-89.4)	2.4%[1,b] (1.4-3.4)	1.6%[1,a] (0.8-2.3)	8.7%[1,2,3] (7.0-10.5)	$P < 0.001$
Modified AdmSCr	86.6% (84.4-88.7)	2.4%[1,b] (1.4-3.4)	2.2%[1,b] (1.3-3.1)	8.8%[1,2,3] (7.0-10.6)	$P < 0.001$
	$P = 0.108$	$P = 0.007$	$P = 0.001$	$P = 0.814$	

C pROCK

	No AKI	Stage 1	Stage 2	Stage 3	
Schwartz	86.8% (84.6-88.9)	7.0%[1] (5.4-8.6)	3.2%[1,2] (2.1-4.3)	3.0%[1,2] (1.9-4.1)	$P < 0.001$
Normsmax	91.1%[a] (89.4-93.0)	4.2%[1,a] (2.9-5.4)	2.2%[1,2] (1.3-3.1)	2.5%[1,2] (1.5-3.5)	$P < 0.001$
AdmSCr	93.2%[a] (91.6-94.8)	4.6%[1,a] (3.3-5.9)	1.6%[1,2,a] (0.8-2.3)	0.6%[1,2,3,a,b] (0.1-1.1)	$P < 0.001$
Modified AdmSCr	89.4%[c] (87.4-91.3)	5.2%[1] (3.8-6.6)	2.4%[1,2] (1.4-3.4)	3.0%[1,2,b,c] (1.9-4.1)	$P < 0.001$
	$P < 0.001$	$P = 0.030$	$P = 0.113$	$P = 0.001$	
