# Supplementary material for: Comparison of diagnostic criteria for acute kidney injury in critically ill children: a multicenter cohort study
Source: Crit Care. 2022 Jul 7;26:207. doi: 10.1186/s13054-022-04083-0 (PMC9264539; doi:10.1186/s13054-022-04083-0)
Supplement: Supplementary file 3 [file 13054_2022_4083_MOESM3_ESM.docx]

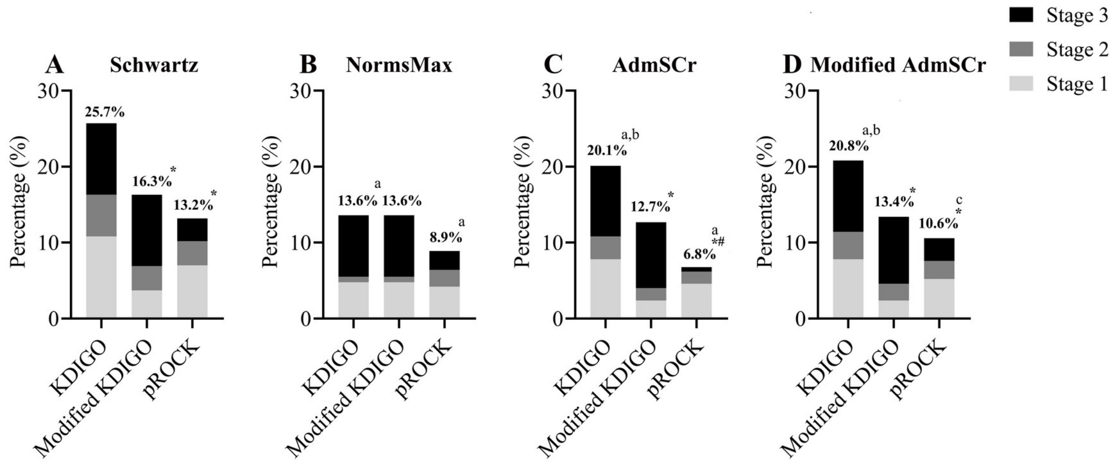

Stage 3
Stage 2
Stage 1
A Schwartz
B NormsMax
C AdmSCr
D Modified AdmSCr
Percentage (%)
0
10
20
30
25.7%
16.3%*
13.2%*
13.6% 13.6%
a
8.9%
a
20.1%
a,b
12.7%*
6.8%
a
*#
20.8%
a,b
13.4%*
10.6%
c
*
KDIGO
Modified KDIGO
pROCK
