# Supplementary material for: Comparison of diagnostic criteria for acute kidney injury in critically ill children: a multicenter cohort study
Source: Crit Care. 2022 Jul 7;26:207. doi: 10.1186/s13054-022-04083-0 (PMC9264539; doi:10.1186/s13054-022-04083-0)
Supplement: Supplementary file 4 [file 13054_2022_4083_MOESM4_ESM.docx]

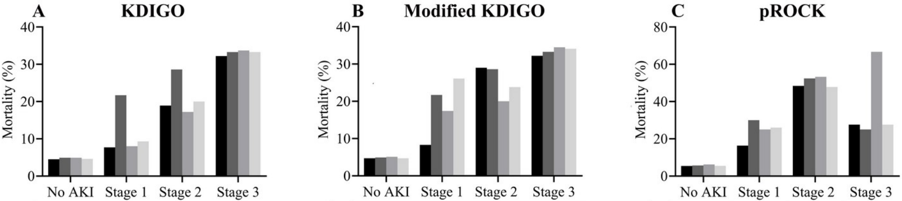

A KDIGO

	No AKI	Stage 1	Stage 2	Stage 3	
Schwartz	4.5% (3.0-6.0)	7.7% (2.5-12.9)	18.9%[1,2] (8.0-29.8)	32.2%[1,2] (22.4-42.1)	$P < 0.001$
Normsmax	4.9% (3.5-6.0)	21.7%[1,a] (9.4-34.1)	28.6%[1] (0-73.7)	33.3%[1] (22.6-44.0)	$P < 0.001$
AdmSCr	4.9% (3.4-6.5)	8.0% (1.7-14.3)	17.2%[1] (2.6-31.9)	33.7%[1,2] (23.7-43.7)	$P < 0.001$
Modified AdmSCr	4.6% (3.1-6.1)	9.3% (2.6-16.1)	20.0%[1] (6.1-33.9)	33.3%[1,2] (23.4-43.3)	$P < 0.001$
	$P = 0.963$	$P = 0.050$	$P = 0.923$	$P = 0.997$	

B Modified KDIGO

	No AKI	Stage 1	Stage 2	Stage 3	
Schwartz	4.7% (3.3-6.2)	8.3% (0-17.8)	29.0%[1,2] (12.1-46.0)	32.2%[1,2] (22.4-42.1)	$P < 0.001$
Normsmax	4.9% (3.5-6.4)	21.7%[1] (9.4-34.1)	28.6%[1] (0-73.7)	33.3%[1] (22.6-44.0)	$P < 0.001$
AdmSCr	5.1% (3.6-6.6)	17.4%[1] (0.6-34.2)	20.0%[1] (0-42.9)	34.5%[1] (24.1-44.9)	$P < 0.001$
Modified AdmSCr	4.7% (3.2-6.1)	26.1%[1] (6.7-45.5)	23.8%[1] (3.9-43.7)	34.1%[1] (23.8-44.4)	$P < 0.001$
	$P = 0.974$	$P = 0.290$	$P = 0.918$	$P = 0.989$	

C pROCK

	No AKI	Stage 1	Stage 2	Stage 3	
Schwartz	5.4% (3.6-6.9)	16.4%[1] (7.3-25.5)	48.4%[1,2] (29.8-67.0)	27.6%[1] (10.3-44.9)	$P < 0.001$
Normsmax	5.7% (4.2-7.2)	30.0%[1] (15.2-44.8)	52.4%[1] (29.1-75.7)	25.0%[1] (6.3-43.7)	$P < 0.001$
AdmSCr	6.2% (4.2-7.8)	25.0%[1] (11.7-38.3)	53.3%[1] (24.7-81.9)	66.7%[1] (12.5-100.0)	$P < 0.001$
Modified AdmSCr	5.5% (3.9-7.0)	26.0%[1] (13.4-38.6)	47.8%[1] (25.7-69.9)	27.6%[1] (10.3-44.9)	$P < 0.001$
	$P = 0.866$	$P = 0.384$	$P = 0.979$	$P = 0.229$	
