# Supplementary material for: Comparison of diagnostic criteria for acute kidney injury in critically ill children: a multicenter cohort study
Source: Crit Care. 2022 Jul 7;26:207. doi: 10.1186/s13054-022-04083-0 (PMC9264539; doi:10.1186/s13054-022-04083-0)
Supplement: Supplementary file 5 [file 13054_2022_4083_MOESM5_ESM.docx]

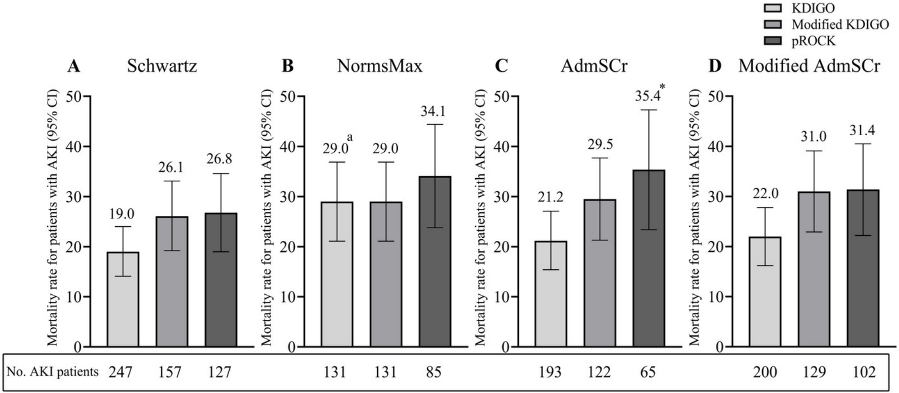
KDIGO
Modified KDIGO
pROCK
A Schwartz
B NormsMax
C AdmSCr
D Modified AdmSCr
Mortality rate for patients with AKI (95% CI)
0
10
20
30
40
50
19.0
26.1
26.8
29.0 a
29.0
34.1
21.2
29.5
35.4 *
22.0
31.0
31.4
No. AKI patients
247
157
127
131
131
85
193
122
65
200
129
102
