# Supplementary material for: Comparison of diagnostic criteria for acute kidney injury in critically ill children: a multicenter cohort study
Source: Crit Care. 2022 Jul 7;26:207. doi: 10.1186/s13054-022-04083-0 (PMC9264539; doi:10.1186/s13054-022-04083-0)

KDIGO

		AOR (95% CI)	*P* value
Schwartz	All AKI Stages	1.96 (1.11-3.47)	0.021
	Stage 1	1.09 (0.44-2.71)	0.848
	Stage 2	2.69 (1.04-6.94)	0.041
	Stage 3	2.84 (1.39-5.82)	0.004
	Stage 2 and 3	2.60 (1.38-4.88)	0.003
NormsMax	All AKI Stages	2.44 (1.31-4.53)	0.005
	Stage 1	2.55 (1.06-6.14)	0.037
	Stage 2	3.21 (0.27-38.24)	0.357
	Stage 3	2.42 (1.15-5.10)	0.020
	Stage 2 and 3	2.42 (1.17-5.02)	0.018
AdmSCr	All AKI Stages	2.17 (1.23-3.84)	0.007
	Stage 1	1.31 (0.50-3.44)	0.588
	Stage 2	2.98 (0.94-9.50)	0.064
	Stage 3	2.60 (1.29-5.24)	0.008
	Stage 2 and 3	2.67 (1.42-5.05)	0.002
Modified-AdmSCr	All AKI Stages	2.25 (1.26-4.02)	0.006
	Stage 1	1.31 (0.50-3.44)	0.579
	Stage 2	3.04 (1.09-8.49)	0.033
	Stage 3	3.01 (1.46-6.20)	0.003
	Stage 2 and 3	2.97 (1.56-5.64)	0.001

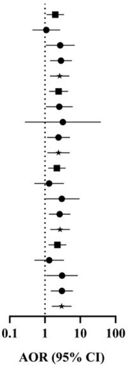

Modified KDIGO

		AOR (95% CI)	*P* value
Schwartz	All AKI Stages	2.49 (1.35-4.57)	0.003
	Stage 1	0.90 (0.21-3.85)	0.891
	Stage 2	4.67 (1.65-13.24)	0.004
	Stage 3	2.75 (1.36-5.56)	0.005
	Stage 2 and 3	3.00 (1.59-5.66)	0.001
NormsMax	All AKI Stages	2.44 (1.31-4.53)	0.005
	Stage 1	2.55 (1.06-6.14)	0.037
	Stage 2	3.21 (0.27-38.24)	0.357
	Stage 3	2.42 (1.15-5.10)	0.020
	Stage 2 and 3	2.42 (1.17-5.02)	0.018
AdmSCr	All AKI Stages	2.83 (1.52-5.24)	0.001
	Stage 1	3.32 (0.97-11.4)	0.057
	Stage 2	3.09 (0.68-14.04)	0.144
	Stage 3	2.57 (1.27-5.19)	0.008
	Stage 2 and 3	2.68 (1.38-5.19)	0.004
Modified-AdmSCr	All AKI Stages	3.28 (1.75-6.15)	<0.001
	Stage 1	4.87 (1.46-16.29)	0.010
	Stage 2	3.16 (0.91-11.00)	0.071
	Stage 3	2.95 (1.42-6.09)	0.004
	Stage 2 and 3	3.02 (1.54-5.89)	0.001

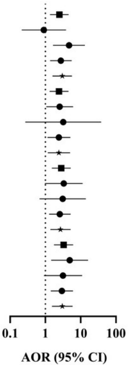

pROCK

		AOR (95% CI)	*P* value
Schwartz	All AKI Stages	2.44 (1.31-4.55)	0.005
	Stage 1	2.25 (1.01-5.03)	0.048
	Stage 2	4.02 (1.51-10.75)	0.005
	Stage 3	1.95 (0.58-6.58)	0.284
	Stage 2 and 3	2.70 (1.18-6.17)	0.018
NormsMax	All AKI Stages	2.64 (1.32-5.26)	0.006
	Stage 1	2.92 (1.18-7.26)	0.021
	Stage 2	3.22 (1.00-10.37)	0.050
	Stage 3	1.80 (0.52-6.19)	0.353
	Stage 2 and 3	2.28 (0.92-5.67)	0.075
AdmSCr	All AKI Stages	3.27 (1.58-6.77)	0.001
	Stage 1	2.46 (1.02-5.92)	0.045
	Stage 2	4.45 (1.11-17.87)	0.036
	Stage 3	14.96 (1.62-138.38)	0.017
	Stage 2 and 3	6.16 (1.83-20.74)	0.003
Modified-AdmSCr	All AKI Stages	2.71 (1.39-5.26)	0.003
	Stage 1	2.99 (1.32-6.79)	0.009
	Stage 2	3.43 (1.09-10.83)	0.036
	Stage 3	2.25 (0.71-7.20)	0.170
	Stage 2 and 3	2.54 (1.06-6.13)	0.037

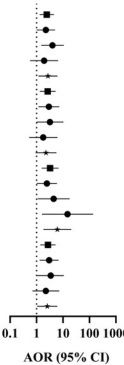

Supplement: Supplementary file 6 [file 13054_2022_4083_MOESM6_ESM.docx]
